# Impacts of canine distemper virus infection on the giant panda population from the perspective of gut microbiota

**DOI:** 10.1038/srep39954

**Published:** 2017-01-04

**Authors:** Na Zhao, Meng Li, Jing Luo, Supen Wang, Shelan Liu, Shan Wang, Wenting Lyu, Lin Chen, Wen Su, Hua Ding, Hongxuan He

**Affiliations:** 1 National Research Center for Wildlife-Borne Diseases, Institute of Zoology, Chinese Academy of Sciences, No. 1-5 Beichenxi Road, Chaoyang District, Beijing, 100101, People’s Republic of China; 2 University of Chinese Academy of Sciences, No. 1-5 Beichenxi Road, Chaoyang District, Beijing, 100101, People’s Republic of China; 3 Zhejiang Provincial Centre for Disease Control and Prevention, Hangzhou, Zhejiang Province, 310051, People’s Republic of China; 4 Hangzhou Centre for Disease Control and Prevention, Hangzhou, Zhejiang Province, 310051, People’s Republic of China

## Abstract

The recent increase in infectious disease outbreaks has been directly linked to the global loss of biodiversity and the decline of some endangered species populations. Between December 2014 and March 2015, five captive giant pandas died due to canine distemper virus (CDV) infection in China. CDV has taken a heavy toll on tigers and lions in recent years. Here, we describe the first gut microbiome diversity study of CDV-infected pandas. By investigating the influence of CDV infection on gut bacterial communities in infected and uninfected individuals and throughout the course of infection, we found that CDV infection distorted the gut microbiota composition by reducing the prevalence of the dominant genera, *Escherichia* and *Clostridium*, and increasing microbial diversity. Our results highlight that increases in intestinal inflammation and changes in the relative abundances of pathogen-containing gut communities occur when individuals become infected with CDV. These results may provide new insights into therapeutics that target the microbiota to attenuate the progression of CDV disease and to reduce the risk of gut-linked disease in individuals with CDV. In addition, our findings underscore the need for better information concerning the dynamics of infection and the damage caused by pathogens in panda populations.

Previous studies have suggested that threats to biodiversity can be attributed to numerous factors, including habitat loss and overexploitation, species invasion and climate change. Notably, a large number of infectious diseases have emerged in wildlife, including the infectious disease chytridiomycosis in amphibians^1^,^2^ and the infectious facial tumor pandemic in Tasmanian devils^3^. The decline and reduced fitness of threatened mammals driven by infectious disease has become an emerging factor that may pose significant risk to endangered species^4^,^5^,^6^. Most of the pathogens that have been identified as causing decline are characterized by a broad host range, high mutation rate and high incidence and fatality rates.

Canine distemper virus (CDV) is a ssRNA virus of the genus *Morbillivirus* that exhibits a high mutation rate and considerable evolutionary potential for host switching. CDV also has a broad host range, including several carnivore species and non-carnivore species such as peccaries and non-human primates. CDV poses challenges for controlling disease in multispecies communities and has led to a massive decline in many wildlife species, including African wild dogs, African lions, Caspian seals and bat-eared foxes^7^,^8^,^9^. Moreover, CDV induces profound short- and long-term immune suppression and impairs immune function by causing the loss of lymphocytes. The reduction of CD4+ T cells is especially prominent during acute CDV infection; these cells help regulate the growth of bacteria within the gut^10^,^11^. In healthy individuals, a principal function of the intestinal bacteria is to protect the gut against exogenous pathogens and potentially harmful indigenous enteropathogens by educating the defensive immune system^12^,^13^. Conversely, distinct changes in this complex microbiota community, such as the breakdown of the normal microbial community, increase disease risk factors, including those for chronic inflammatory disorders^14^, metabolic disorders^15^, cardiovascular diseases^16^, respiratory system diseases^17^, and liver diseases^18^.

The giant panda (*Ailuropoda melanoleuca*), which is listed as an endangered species by the International Union for Conservation of Nature (IUCN), is one of the most adored and protected rare species around the world. Due to governmental policy and rapidly advancing conservation science, pandas experienced obvious population growth over recent decades. According to the latest Fourth National Giant Panda Survey, the proportion of suitable habitat area is expanding (up 11.8% in the past decade), although 1864 wild giant pandas (up 16.4%) and 375 captive pandas (up 128.66%) live in fragmented habitats and conservation breeding programs in China^19^. Here, we report on the CDV outbreak in giant pandas that occurred between December 2014 and March 2015 in Shaanxi Rare Wildlife Rescue and Breeding Research Center, China^20^,^21^. In this outbreak, five giant pandas died due to CDV infection (22.7% mortality). In addition, CDV-positive individuals suffer from an increased incidence of diarrhea^21^ in the absence of obvious enteric pathogens. As the most intriguing herbivorous mammalian species in the family *Ursidae*, pandas are well known as an evolutionarily distinct bamboo specialist, and yet, this species exhibits a gastrointestinal tract typical of carnivores. Its gut microbiota is dominated by the genera *Escherichia* and *Streptococcus*^22^,^23^. Considering the potential relationship between viral infection and the gut microbiome^17^,^24^,^25^ and the vital role that the immune system plays in regulating the gut microbiome^26^,^27^, understanding how the composition of the microbiota changes as a result of CDV infection and the implications of these changes on the prevalence of disease-causing pathogens is vitally important.

To evaluate the role of CDV infection in shaping the gut microbiome, to determine how CDV affects the content and stability of the gut microbiome in pandas, and to understand the role and influence of CDV dynamics in this outbreak, we collected fecal samples from four individuals who were infected with CDV as well as from six healthy individuals and followed the gut bacterial community of two CDV-positive individuals (GP-603 and GP-754). We were thus able to investigate alteration of the gut microbiome in individuals with or without CDV infection and to track the composition of the gut microbiota as the disease progressed in infected individuals.

## Results

### Characteristics of the individuals and 16 S rRNA gene sequence data

We sequenced the V3-V4 region of the 16S rRNA genes in sixteen fecal samples collected from CDV-infected individuals (n = 4), including ten CDV-positive fecal samples collected during different stages of the infection and six collected from healthy controls. After quality trimming and chimera detection, an average of 36,014 high-quality sequences (range from 29,627 to 39,425) remained, and after sample rarefaction, these sequences were clustered into 178 operational taxonomic units (OTUs) at a threshold of 97% sequence identity. Complete sample information and sequencing results are presented in Table 1.

**Figure 1:**
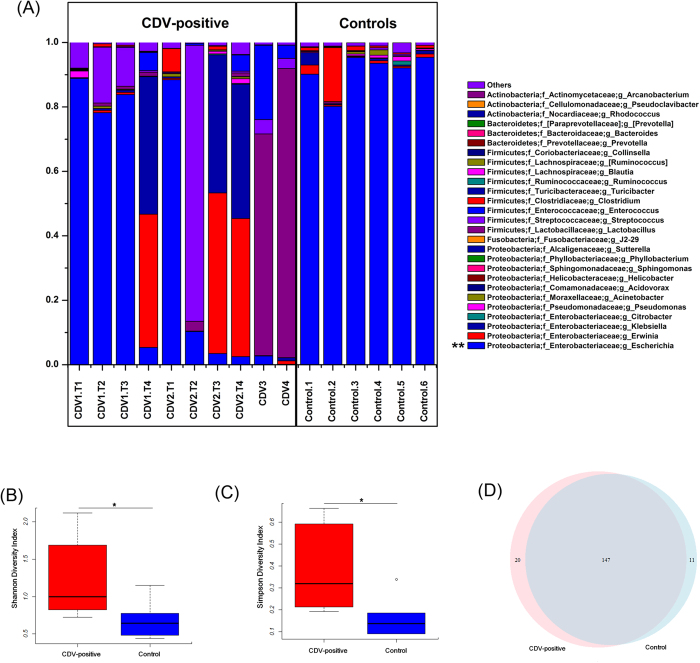
Comparison of the gut microbiota composition between CDV-infected individuals and healthy controls. (**A**) Relative contribution of the top 20 dominant genera in each sample. Genera that differed significantly between the CDV-infected individuals and the healthy controls are marked with asterisks (P < 0.01 using one-way ANOVA). The Shannon (**B**) and Simpson (**C**) indices were used to estimate diversity (data shown as the mean ± SEM). A Venn diagram illustrating the overlap of OTUs in the gut microbiota between individuals with or without CDV infection (**D**).

In total, sequences from the fecal microbiota could be classified into 74 genera: 56 genera in healthy controls, and 70 genera in CDV-positive samples. Each of the 20 predominant genera showed wide variation in abundance across the collected samples (Fig. 1A). In particular, the abundance of *Escherichia*, the dominant genus, differed significantly between the CDV-positive samples and the healthy control samples (*P* < 0.01).

### CDV infection destabilizes the composition of the fecal microbiota

An influence of CDV infection on gut microbial communities has not been previously reported. To further determine whether CDV infection altered the composition of the gut microbiota, we compared the diversity and richness indices between the two groups. The fecal microbiota of CDV-infected individuals displayed greater overall diversity, with greater evenness and richness, as measured by the Shannon (*P* = 0.036; Fig. 1B, Supplementary Table S1) and Simpson diversity indices (*P* = 0.018; Fig. 1C, Supplementary Table S1).

In contrast to the diversity indices, however, there were no significant differences (*P* > 0.05) in the richness indices (Chao 1 and observed species) between the two groups (Supplementary Fig. S1, Supplementary Table S1). The rarefaction curves showed that the total richness of the microbial community had been sampled completely. To better understand the differences in richness between the two groups, the overlap between the groups was illustrated using a Venn diagram. This analysis showed that the virus infection samples and healthy samples contained 20 and 11 unique OTUs, respectively (Fig. 1D).

**Figure 2:**
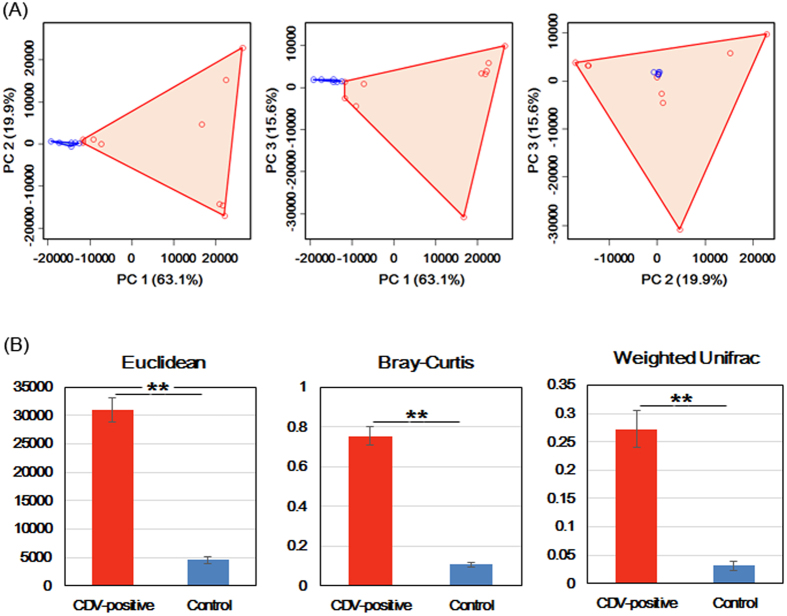
Greater compositional heterogeneity among CDV-infected gut microbiomes than among the uninfected controls. (**A**) The difference in the composition of the gut microbiota is illustrated by principal-coordinate plots of the Euclidean distances among samples. All pairwise comparisons involved the first, second and third principal axes. The first, second and third principal axes explained most of the variance (63.1%, 19.9% and 15.6%, respectively). Dots and surrounding areas correspond to gut bacterial communities from the healthy controls (blue) and the CDV-infected individuals (red). (**B**) The relative abundance of bacterial phylotypes between the CDV-infected samples (red bars) and the healthy control samples (blue bars). Asterisks indicate significant differences (P < 0.01), and the error bars denote 95% confidence intervals for the mean values.

To measure the degree to which the gut microbiota of the CDV-infected individuals differed from the microbiota of the healthy controls, a principal coordinate analysis (PCoA) was conducted based on the Euclidean distance between the fecal samples. This analysis showed a strong difference in the microbiota of individuals with CDV infection compared to that of healthy controls. The composition of the fecal microbiota of the CDV-infected individuals differed significantly from that of the fecal microbiota of the healthy controls, and community composition was significantly more variable in the samples from the CDV-infected individuals than in the healthy control samples (*P* < 0.01; Fig. 2A).

Euclidean distance metrics, Bray-Curtis dissimilarity metrics, and weighted UniFrac distance metrics showed that in terms of changes in frequency, relative abundance and the presence or absence of bacterial phylotypes, the communities of the CDV-infected individuals were less stable than those of the healthy controls (*P* < 0.01; Fig. 2B).

### CDV-associated alterations in fecal microbiota

**Figure 3:**
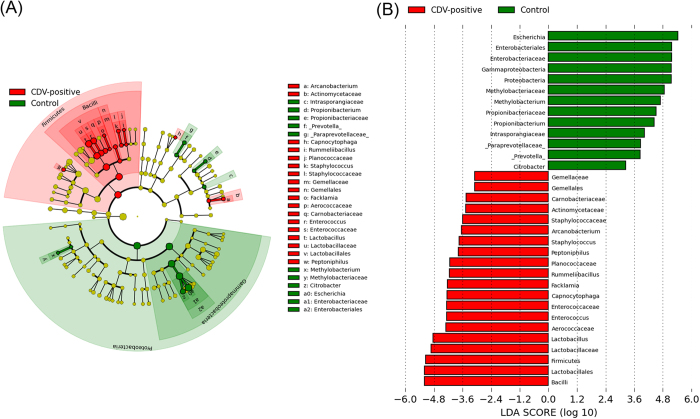
The taxa whose abundances differed between the CDV-infected individuals and the healthy controls were identified by LEfSe. (**A**) Taxonomic cladogram obtained from LEfSe sequence analysis (relative abundance ≥0.5%). Biomarker taxa are highlighted by colored circles and shaded areas (CDV-positive samples are shown in red and healthy control samples are shown in blue). Each circle’s diameter reflects the abundance of that taxa in the community. (**B**) The taxa whose abundance differed between the CDV-infected samples (red bars) and the healthy control samples (blue bars). The cutoff value of ≥2.0 used for the linear discriminant analysis (LDA) is shown.

To identify the specific bacterial taxa associated with CDV infection, we compared the fecal microbiota of healthy controls and CDV-infected individuals using the linear discriminant analysis (LDA) effect size (LEfSe) method. A cladogram representative of the structure of the fecal microbiota and the predominant bacteria is shown in Fig. 3A and B; the greatest differences in taxa between the two communities are displayed. The relative abundance of the *Firmicutes* phylum (Supplementary Fig. S2A) was significantly higher (LDA > 2) in the CDV-positive group compared with the healthy controls, while the relative abundance of the *Proteobacteria* phylum (Supplementary Fig. S2B) was significantly lower (LDA > 2) in the CDV-positive group compared with the healthy controls. Moreover, consistent with alpha diversity indices such as the Shannon index, clustering analysis of the top 20 genera highlighted differences in their distributions due to CDV infection. Clearly, the aberrant composition of the fecal microbiota was associated with CDV infection (Supplementary Fig. S3).

### CDV infection increases the frequency of CD-associated bacterial genera within the gut microbiota

**Figure 4:**
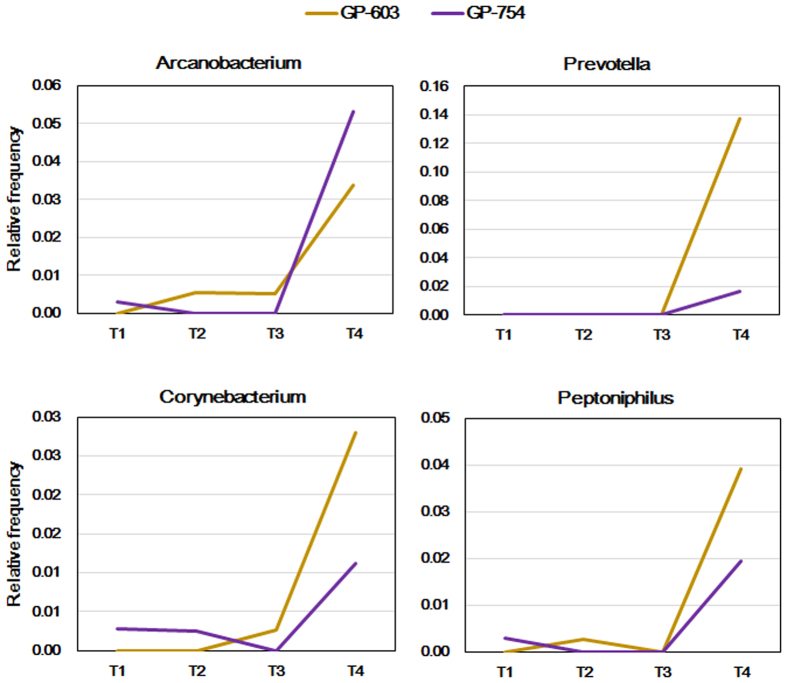
Increase in the frequency of bacteria from disease-associated genera in the CDV-infected individuals. Relative abundance of Arcanobacterium, Prevotella, Corynebacterium and Peptoniphilus in the gut microbiota of GP-603 and GP-754 at different time points as the infection progressed. The longitudinal samples (TN) shown in Fig. 4 match the dates of sampling listed in Table 1.

In the fecal samples collected from CDV-infected individuals, spikes in the relative abundance of pathogen-containing gut communities were observed; such spikes were absent from the healthy control samples. Moreover, the genera *Arcanobacterium, Corynebacterium, Peptoniphilus* and *Prevotella* exhibited spikes in abundance when pandas were infected with CDV, especially during the late stages of infection (Fig. 4). These genera contain opportunistic pathogens and were never detected at high abundances in the six healthy pandas surveyed previously.

### CDV infection increases systemic and local intestinal inflammation

**Figure 5:**
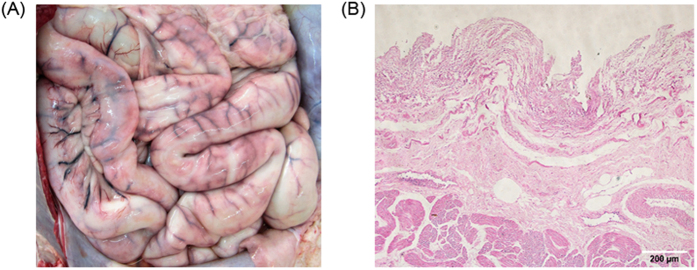
Intestinal clinical signs and pathological changes among the CDV-infected giant pandas, China, 2014. CDV-infected pandas showed signs of (**a**) severe enteritis with intestinal hemorrhage of the serosa and (**b**) necrosis of the intestine. Fixed tissue samples were stained with hematoxylin and eosin (original magnification X100, scale bar = 200 μm).

CDV-positive individuals experience an increased incidence of diarrhea^21^ in the absence of obvious enteric pathogens and severe enteritis with intestinal hemorrhage of the serosa and necrosis of the intestine (Fig. 5). Moreover, CDV can be identified in multiple organs, including the intestine, by RT-PCR (Supplementary Fig. S4).

**Figure 6:**
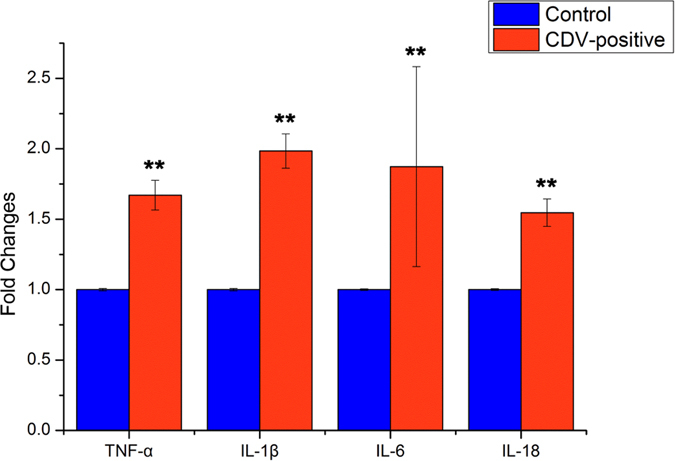
CDV infection modulates cytokine release and increases the systemic inflammatory response. Quantitative RT-PCR analysis of the expression of pro-inflammatory TNF-α, IL-1β, IL-6 and IL-18 in plasma from giant pandas at day 7 post CD onset is shown as fold change ± SEM. Asterisks indicate statistically significant differences (P < 0.01 by one-way ANOVA), and the error bars denote 95% confidence intervals for the mean values.

Therefore, we expected an expansion of pro-inflammatory cytokines and CD-associated bacteria with CD disease. Due to the absence of healthy intestine samples, we could not directly compare the local cytokine levels of the intestines between the two groups. Therefore, we compared the production of pro-inflammatory cytokines in the peripheral blood, which was related to systemic inflammation. The pro-inflammatory cytokines tumor necrosis factor α (TNF-α), interleukin-1β (IL-1β), IL-6 and IL-18 were increased in the plasma samples from CDV-positive individuals at day 7 post-CD onset. Additionally, we observed 1.5-fold to 2-fold upregulation of pro-inflammatory cytokines in the CDV-positive pandas compared to that in the healthy controls (*P* < 0.01, Fig. 6).

## Discussion

In this outbreak, CDV produced an acute febrile and highly fatal disease in captive giant pandas in China. Due to the efforts of the Chinese government and the State Forestry Administration, the virus has not infected the other pandas in the breeding center and has not spread to other reserves or to wild pandas on the other side of the Qin Mountains from the center. This study represents the first use of high-throughput sequencing of the V3-V4 region of the 16S rRNA genes to analyze changes in the gut microbiota in CDV-infected pandas and provides a new perspective on the influence of infectious diseases in panda conservation. Microbiota community changes were evaluated in 80% (4/5) of the CDV-positive pandas in this outbreak and were compared to changes in healthy controls. We also separately tracked the composition of the gut microbiota of two infected individuals (GP-603 and GP-754) as the infection progressed. We found evidence that the microbiota began to change early during the infection and continued to change over time.

In this study, we demonstrated a significant perturbation of the gut microbiome of pandas due to CDV infection. This perturbation was characterized by a reduction in the dominance of *Escherichia* and *Clostridium* (Fig. 1A) and an accompanying increase in microbial diversity (Fig. 1B,C), similar to the increased diversity that has been observed in SIV/HIV-infected individuals^28^,^29^,^30^. In addition, the gut microbiota of the CDV-infected samples occupied a greatly expanded area of compositional space that was never accessed by the gut communities of the uninfected individuals (Fig. 2A); this observation also parallels an effect of SIV infection in chimpanzees^29^. Previous studies have demonstrated that infection with immunosuppressive viruses, such as SIV infection in primates and HIV infection in humans, may provide a link between gut microbial communities and the progression of AIDS^29^,^30^,^31^,^32^. As another immunosuppressive animal virus, CDV infects lymphocytes and monocytes and produces severe leukopenia, including reducing the number of lymphocytes (both B and T cells) in the circulation and lymphoid tissues. CD4+ T cells have been reported to help regulate the growth of bacteria within the gut microbiome^33^. Furthermore, T cells and Toll-like receptor 4 (TLR-4) on B cells play indispensable roles in the generation of microbiota-specific immunoglobulin G (IgG) and in increasing resistance to systemic infection^12^. To some extent, the changes in the gut microbiota support the hypothesis that the immune system plays an important role in shaping the gut bacterial composition^34^,^35^,^36^,^37^. However, due to the failure to isolate and culture lymphocytes from the fresh blood of giant pandas at the time of the outbreak, we cannot conduct an immune response experiment in giant panda lymphocytes. Thus, no explicit evidence has shown that CDV infection influences the gut microbiota through the immunosuppressive ability of the virus.

Furthermore, increased intestinal inflammation (Fig. 5) and systemic pro-inflammatory cytokine levels were evaluated in CDV-positive individuals (Fig. 6). Expansion of the pro-inflammatory cytokines TNF-α, IL-1β, IL-6 and IL-18 in the peripheral blood presumably is important for the immune responses of infectious individuals^38^. In many mammalian studies (including human, pig and mouse models) investigating aging, bacterial pathogen infections, obesity, depressive disorders and gut diseases, correlations have been made between the gut microbiota and the pro-inflammatory cytokine levels in plasma samples^39^,^40^,^41^,^42^,^43^,^44^,^45^,^46^. Considering the underlying correlations, the pro-inflammatory markers observed in the present study may be related to the dysbiotic microbiota in CDV-infectious pandas. This observation of intestinal inflammation and the systemic pro-inflammatory cytokine levels suggest that the CDV-related differences in the gut microbiota compositions may be affected by not only the local but also the systemic inflammatory status. Unfortunately, due to the absence of healthy intestines, we could not directly compare the local inflammatory cytokines in the intestines of the pandas.

Notably, the relative frequencies of some bacteria, including the genera *Prevotella, Peptoniphilus, Arcanobacterium* and *Corynebacterium*, present within two individuals (GP-603 and GP-754) increased in abundance as the infection progressed, and this increase was particularly evident in the final sample collected before the deaths of these individuals (Fig. 4). These genera contain opportunistic pathogens^47^,^48^,^49^,^50^ and were not detected in the six healthy controls. It will be important to determine whether the CDV infection-induced increase in the frequency of disease-associated genera could serve as an early predictor and pose health risks to hosts.

In general, the novel findings of the present study suggested that CDV infection disturbed the gut bacterial composition in pandas. This study of CDV-infected giant pandas will help elucidate the role of CDV infection in shaping the composition of the gut microbiota and altering gut inflammation. Thus, new insights may be provided into therapeutics that target the microbiota to attenuate the progression of CDV disease and to reduce the risk of gut-linked disease in individuals with CDV. Alternatively, opportunistic pathogens might be the source of the increase in unique phylotypes in the fecal microbiota of CDV-infected individuals.

Over the past two decades, due to the Chinese government’s commitment and visionary policies and management actions as well as to a remarkable increase in the biological and ecological knowledge of pandas, the panda population has rapidly expanded. This CDV outbreak in pandas highlighted the impact of infectious diseases on the conservation of this iconic species. To date, the mode and/or source of CDV transmission in pandas throughout this outbreak remain unclear. Due to host coevolution and other driving factors, such as climate change and overexploitation, many pathogens have evolved numerous strategies to evade the immune response of the host. Our findings underscore the need for better information concerning the distribution and impacts of infectious diseases in panda populations.

This study is subject to limitations. Firstly, not all CDV-infected individuals in this outbreak were enrolled in this study. Secondly, we failed to isolate and culture lymphocytes from the fresh blood of CDV-positive individuals. Thus, we were not able to explicitly correlate the immune decline with the gut microbiota composition in the CDV-infected giant pandas. Finally, we could not compare the local cytokine levels in the intestines between the CDV-positive individuals and the healthy controls. In future studies using dog or mouse infection models, we will evaluate the effects of CDV (Giant Panda isolate) infection on selected bacteria and the lymphocyte immune response and analyze local inflammation of the intestine to further evaluate the mechanism underlying the gut microbiota changes caused by CDV infection.

## Materials and Methods

### Ethical approval

The research complied with protocols approved by the Animal Care Committee of the Chinese Academy of Sciences and conformed to the regulatory requirements of Shaanxi Rare Wildlife Rescue and Breeding Research Center, Shaanxi Province, China.

### Sample Collection

We describe giant pandas with or without CDV infection within the Shaanxi Rare Wildlife Rescue and Breeding Research Center, Shaanxi Province, China. Two cohorts of giant pandas were selected. The CDV-infected individuals included four giant pandas that were infected with CDV, as confirmed by antigen diagnose and real-time RT-PCR identification^21^. Ten fecal and blood samples were collected as the infection progressed. The healthy controls included six healthy individuals that were matched to the CDV-infected individuals in terms of sex and age (Table 1).

A total of 16 samples from 10 giant pandas were collected between December 2014 and March 2015. Fecal samples were collected immediately after defecation in the early morning and the dates of stools collected (including the repeated samples) were shown in Table 1. The fresh fecal samples were collected from the inside feces into sterile plastic container, snap-frozen in liquid N_2_, stored on dry ice, and delivered to the lab in thirty-minutes, and immediately stored at −80 °C until further analysis. Serum was separated by 20 min spin at 2,000 × g, and the resulting plasma were stored at −80 °C, and used for the cytokines evaluation.

### DNA extraction from feces

The frozen aliquots (200 mg per aliquot) were added to a 2 ml screw-cap and thawed on ice until 1.4 ml ASL buffer from the QIAamp DNA Stool Mini Kit (Qiagen, Hilden, Germany) was added. The subsequent DNA extraction steps were conducted according to the QIAamp Kit protocol. Finally, the DNA was dissolved in 200 μl sterile ddH2O and stored at −20 °C until use. The concentration of the DNA was measured using a NanoDrop™ 2000 (Thermo Scientific), and its molecular size was determined via electrophoresis on 1.0% (wt/vol) agarose gels with 0.5 mg/ml ethidium bromide and UV-light photography.

### 16S rRNA gene amplification

The 16 S rRNA genes were amplified in triplicate using primers (341F: 5′-CCTAYGGGRBGCASCAG-3′, 806R: 5′-GGACTACHVGGGTWTCTAAT-3′) specific for the V3-V4 region of the 16 S rRNA genes. The PCR products were detected using 2% agarose gels, and samples with a bright strip between 400 bp and 450 bp were chosen. Then, the PCR products were purified using the Qiagen Gel Extraction Kit (Qiagen, Germany). Sequencing libraries were generated using the TruSeq DNA PCR-Free Sample Preparation Kit (Illumina, USA) according to the manufacturer’s recommendations, and index codes were added. The quality of the library was assessed using the Qubit®2.0 Fluorometer (Thermo Scientific) and Agilent Bioanalyzer 2100 system. Finally, the library was sequenced on an Illumina Miseq platform, and 250 bp paired-end reads were generated.

### RNA preparation and quantitative RT-PCR

Total RNA was extracted using RNeasy MiniElute Cleanup Kit (Qiagen) according to the manufacturer instructions. The concentration of total RNA was qualified by determining the OD260 value with Nanodrop 2000TM (Nano-Drop Technologies). 2 μg RNA was reverse transcribed with M-MLV reverse transcriptase (Promega). Real-time PCR was performed using the ABI 7500 Fast Real-Time PCR system (Life technologies). All qRT-PCR reactions were prepared in 25 μl with final concentrations of 1× SYBR FastStart Universal SYBR Green Master (Rox) (Roche life science), and 0.2 μM forward and reverse primers, using the following conditions: 95 °C for 10 min; 40 cycles of 95 °C (15 s) and 60 °C (60 s). Direct detection of PCR products was monitored by measuring increase in fluorescence. Reactions were performed in triplicate, Ct values were normalized to GAPDH levels, and fold changes were calculated (ΔΔCt) with respect to the healthy control group. Sequences of primers used for qRT-PCR, which are specific for cytokines of pandas, are shown in Table 2. Results are expressed as mean ± SEM.

### Statistical and bioinformatics analysis

Paired-end reads were assigned to samples based on their unique barcode and truncated by cutting off the barcode and primer sequence. The paired-end reads were merged using FLASH (V1.2.7, http://ccb.jhu.edu/software/FLASH/), and raw tags were successfully spliced. Quality filtering of the raw tags was performed to obtain high-quality clean tags according to the QIIME quality control process (V1.7.0, http://qiime.org/index.html).

Quality was controlled as follows: a. If the average quality score for a 50 bp sliding window was ≤19, then the tags were discarded from the original reads. b. Tags that contained more than 25% low-quality bases were removed.

Sequence analysis was performed with Uparse software (http://drive5.com/uparse/). Sequences with ≥97% similarity were assigned to the same OTUs. A representative sequence for each OTU was screened for further annotation.

For each representative sequence, the GreenGene Database (http://greengenes.lbl.gov/cgi-bin/nph-index.cgi) was used to annotate the taxonomic information based on the RDP classifier (Version 2.2, http://sourceforge.net/projects/rdp-classifier/) algorithm.

The analysis of alpha diversity, which included calculation of the observed species, Chao 1, Shannon, and Simpson indices as well as the beta diversity analysis, which utilized both weighted and unweighted UniFrac, was conducted with QIIME (Version 1.7.0). Cluster analysis was preceded by principal coordinate analysis (PCoA), and the results of all of the analyses were displayed in R software (Version 2.15.3).

## Additional Information

**Accession codes:** The sequencing reads generated from the giant panda individuals described in this study have been deposited in the sequence read archive (SRA) at NCBI under the accession number SRP071134.

**How to cite this article:** Zhao, N. *et al*. Impacts of canine distemper virus infection on the giant panda population from the perspective of gut microbiota. *Sci. Rep.* **7**, 39954; doi: 10.1038/srep39954 (2017).

**Publisher's note:** Springer Nature remains neutral with regard to jurisdictional claims in published maps and institutional affiliations.

## Supplementary Material

Supplementary Information

## Figures and Tables

**Table 1:** Source, Reads and OTU Counts of Fecal samples from Giant Pandas that Became Naturally Infected with CDV and healthy controls.

ID number	DOB†	DOI‡	DOO¶	DOD§	Sex	Sample Name	Collection Date	CDV status	Number of Reads	Number of OTUs
GP-603	08/2005	05/01/2015	10/01/2015	23/01/2015	F	CDV1.T1	05/01/2015	positive	29,627	75
						CDV1.T2	11/01/2015	positive	37,560	41
						CDV1.T3	17/01/2015	positive	38,141	51
						CDV1.T4	23/01/2015	positive	36,204	71
GP-754	08/2009	26/12/2014	02/01/2015	04/02/2015	F	CDV2.T1	26/12/2014	positive	35,520	58
						CDV2.T2	23/01/2015	positive	39,425	26
						CDV2.T3	31/01/2015	positive	38,012	54
						CDV2.T4	04/02/2015	positive	36,368	90
GP-700	/2006	24/12/2014	24/12/2014	04/01/2015	F	CDV3	02/01/2015	positive	31,710	37
GP-753	08/2009	24/12/2014	14/03/2015	08/04/2015	M	CDV4	30/03/2015	positive	37,195	30
GP-757	09/2009	*	*	*	F	Control.1	23/01/2015	negative	37,586	60
GP-500	1985	*	*	*	F	Control.2	23/01/2015	negative	34,396	66
GP-908	18/07/2013	*	*	*	F	Control.3	23/01/2015	negative	37,091	78
GP-803	2004 ± 5	*	*	*	M	Control.4	23/01/2015	negative	33,157	49
GP-753	18/08/2009	*	*	*	M	Control.5	23/01/2015	negative	38,188	53
GP-509	06/08/2000	*	*	*	F	Control.6	23/01/2015	negative	36,039	51

^†^Dates of birth.

^‡^Dates of infection. Asterisks denote giant pandas that are presently CDV-negative.

^¶^Dates of onset. Asterisks denote giant pandas that are presently CDV-negative.

^§^Dates of death. Asterisks denote giant pandas that are presently alive.

**Table 2:** Sequences of specific primers used for qRT-PCR.

Gene	Primer sequence (5′-3′)	Product size (bp)
GAPDH	CTCTGGAAAGCTGTGGCGTGATGATGCCAGTGAGCTTCCCGTTCAG	120
TNF-α	CATCTTCTCGAACTCCGAGTGACAATGGGAGTAGATGAGGTACAGCCCG	175
IL-1β	GAACCAACAAGCGGTGTTCCGCATGGCAGGGTGGGCTTCCCGTCTT	134
IL-6	GATGAGGCCACCTCAAATAGACCCAGTGCCTCCTTGCTGTCTTCACA	141
IL-18	ACTTTGGCAAGCTCGAACCTACAAACACGGGTTGATTTCCCT	90
