# Supplementary material for: Impacts of canine distemper virus infection on the giant panda population from the perspective of gut microbiota
Source: Sci Rep. 2017 Jan 4;7:39954. doi: 10.1038/srep39954 (PMC5209704; doi:10.1038/srep39954)
Supplement: Supplementary Information [file srep39954-s1.pdf]

Supplementary information

Impacts of canine distemper virus infection on the giant panda population from the perspective of gut microbiota

Na Zhao,[1,2,†] Meng Li,[1,2,†] Jing Luo,[1] Supen Wang,[1] Shelan Liu,[3] Shan Wang,[1] Wenting Lyu,[1,2] Lin Chen,[1,2] Wen Su,[1,2] Hua Ding,[4] and Hongxuan He,[1,*]

1. National Research Center for Wildlife-Borne Diseases, Institute of Zoology, Chinese Academy of Sciences, No. 1-5 Beichenxi Road, Chaoyang District, Beijing, People's Republic of China 100101;

2. University of Chinese Academy of Sciences, No. 1-5 Beichenxi Road, Chaoyang District, Beijing, People's Republic of China 100101;

3. Zhejiang Provincial Centre for Disease Control and Prevention, Hangzhou, Zhejiang Province, People's Republic of China 310051

4. Hangzhou Centre for Disease Control and Prevention, Hangzhou, Zhejiang Province, People's Republic of China 310051

† These authors contributed equally to this work.

* Corresponding author

Prof. Hongxuan He, National Research Center for Wildlife-Borne Diseases, Institute of Zoology, Chinese Academy of Sciences, No. 1-5 Beichenxi Road, Chaoyang District, Beijing, People's Republic of China 100101

Tel/Fax: 86-10-64807118(O/F); E-mail: hehx@ioz.ac.cn

(A)

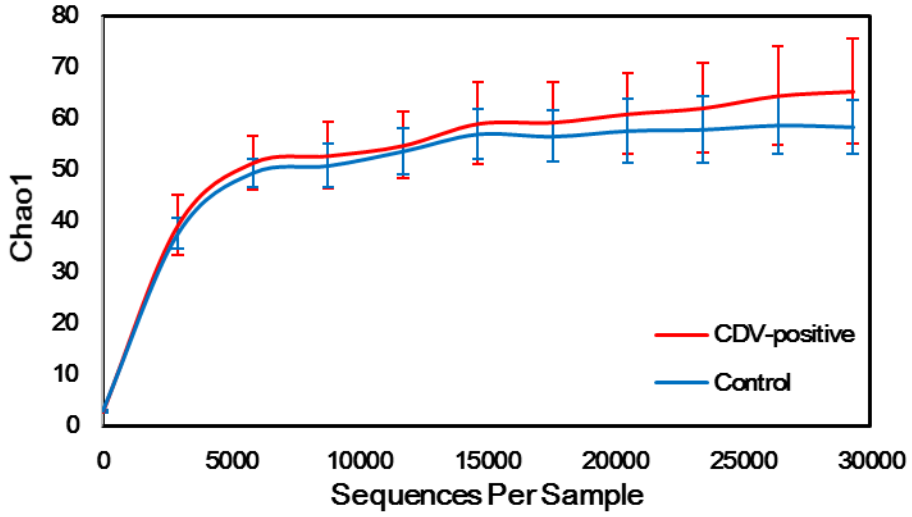

(B)

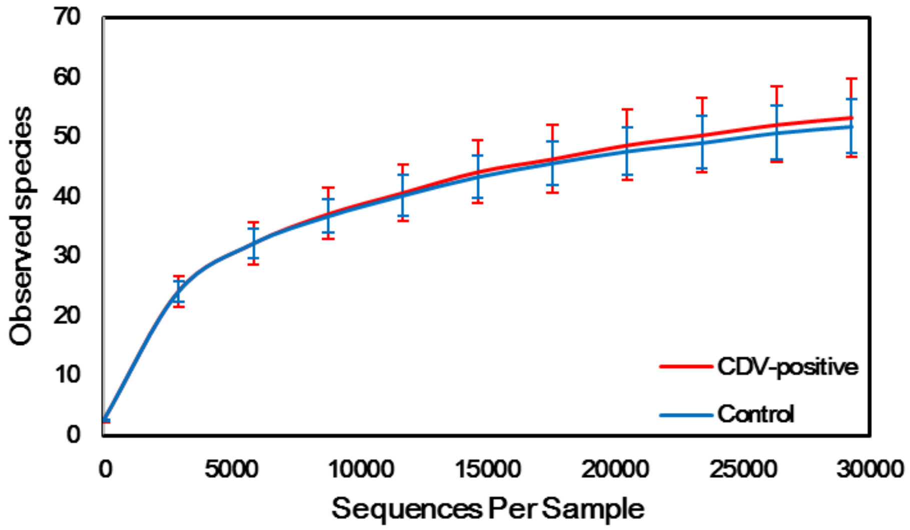

Supplementary Fig. S1. Estimators of richness and diversity used to compare the gut microbiota of the CDV-positive samples and the healthy control samples. (A) The Chao 1 index was calculated from the sequencing data using QIIME (Version 1.7.0) at 97% similarity to estimate the richness. (B) Structural comparison of the two groups. The vertical axis shows the number of OTUs that would be expected to be found after sampling the number of tags or sequences shown on the horizontal axis.

(A)

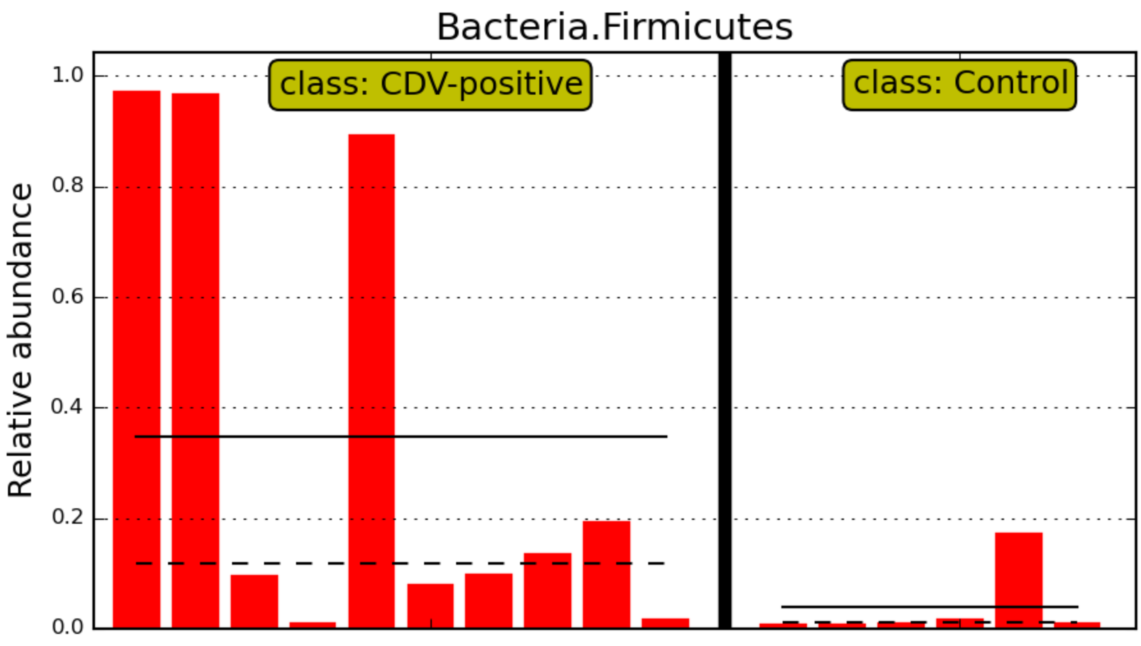

(B)

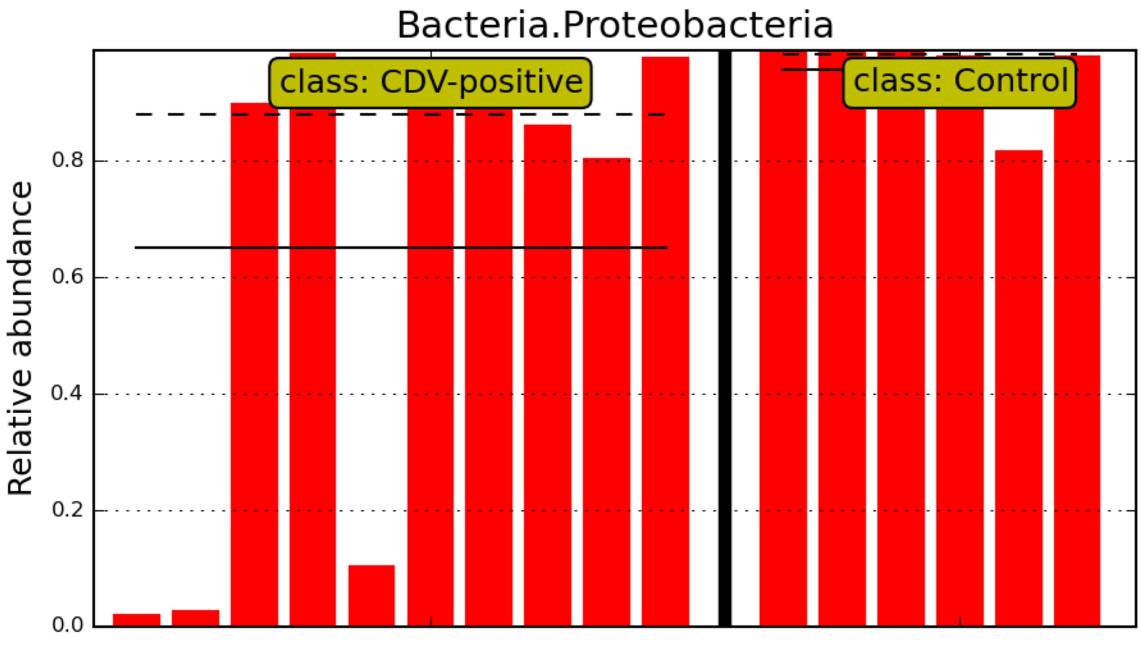

Supplementary Fig. S2. The taxa whose abundance differed between the CDV-infected individuals and the healthy controls. The abundance of the phylum *Firmicutes* (A) increased while the abundance of the phylum *Proteobacteria* (B) decreased in the gut microbiota of CDV-positive individuals.

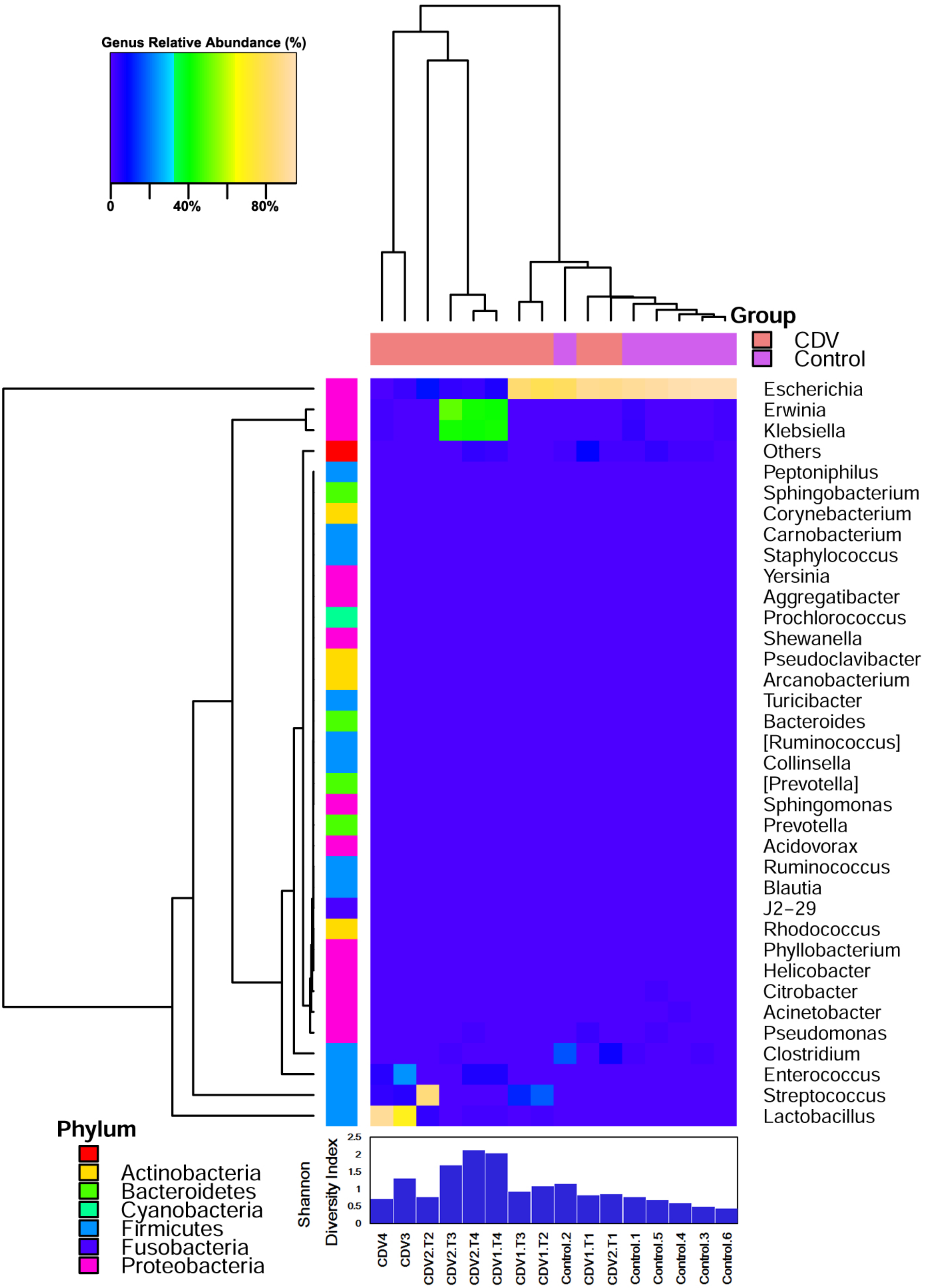
Genus Relative Abundance (%)
0
40%
80%
Group
CDV
Control
Escherichia
Erwinia
Klebsiella
Others
Peptoniphilus
Sphingobacterium
Corynebacterium
Carnobacterium
Staphylococcus
Yersinia
Aggregatibacter
Prochlorococcus
Shewanella
Pseudoclavibacter
Arcanobacterium
Turicibacter
Bacteroides
[Ruminococcus]
Collinsella
[Prevotella]
Sphingomonas
Prevotella
Acidovorax
Ruminococcus
Blautia
J2−29
Rhodococcus
Phyllobacterium
Helicobacter
Citrobacter
Acinetobacter
Pseudomonas
Clostridium
Enterococcus
Streptococcus
Lactobacillus
Phylum
Actinobacteria
Bacteroidetes
Cyanobacteria
Firmicutes
Fusobacteria
Proteobacteria
Shannon Diversity Index
2.5
2
1.5
1
0.5
0
CDV4
CDV3
CDV2.T2
CDV2.T3
CDV2.T4
CDV1.T4
CDV1.T3
CDV1.T2
Control.2
CDV1.T1
CDV2.T1
Control.1
Control.5
Control.4
Control.3
Control.6

Supplementary Fig. S3. Heatmap illustrating genus-level differences between the CDV-infected individuals and the healthy controls. The relative abundance of each genus is indicated by a Z score, and a gradient of color from blue to yellow indicates the abundance, ranging from low to high. The gut microbiota was found to have a distinctive bacterial composition, which was also significantly associated with the Shannon index.

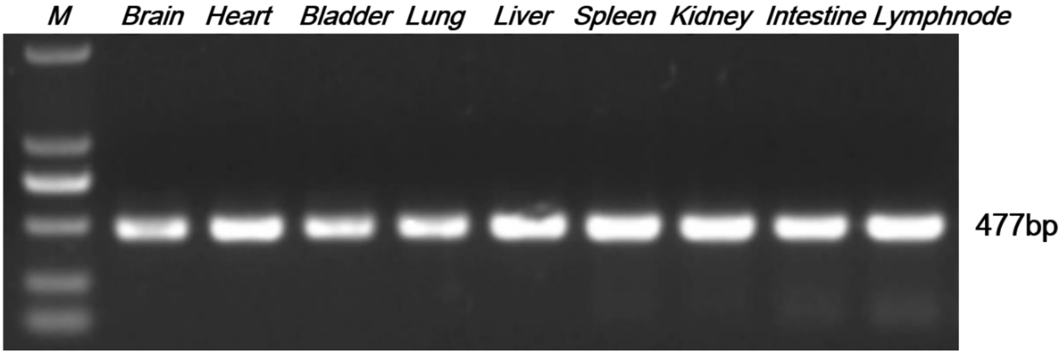

Supplementary Fig. S4. Agarose gel electrophoresis of hemagglutinin (H) gene fragment (477bp) from CDV by RT-PCR. Amplification products from different organs in the first fatal giant panda infected with CDV at day 7 post CD illness onset. Marker (Takara, DL2000): 2000bp, 1000bp, 750bp, 500bp, 250bp, 100bp.

Supplementary Table 1. Comparison of phylotype coverage and diversity index at 97% similarity

Sample name	Chao 1 Average	Observed species Average	Shannon diversity index	Simpson diversity index
CDV-positive	65.39	53.29	1.23	0.39
Controls	66.76	59.5	0.69	0.16
